# A novel sex-determining QTL in Nile tilapia (*Oreochromis niloticus*)

**DOI:** 10.1186/s12864-015-1383-x

**Published:** 2015-03-11

**Authors:** Christos Palaiokostas, Michaël Bekaert, Mohd GQ Khan, John B Taggart, Karim Gharbi, Brendan J McAndrew, David J Penman

**Affiliations:** Institute of Aquaculture, School of Natural Sciences, University of Stirling, Stirling, FK9 4LA Scotland UK; Department of Fisheries Biology and Genetics, Bangladesh Agricultural University, Mymensingh, Bangladesh; Edinburgh Genomics, Ashworth Laboratories, King’s Buildings, University of Edinburgh, Edinburgh, EH9 3JT Scotland UK

**Keywords:** *Oreochromis niloticus*, Sex reversal, QTL mapping, ddRAD-seq, Aquaculture

## Abstract

**Background:**

Fish species often exhibit significant sexual dimorphism for commercially important traits. Accordingly, the control of phenotypic sex, and in particular the production of monosex cultures, is of particular interest to the aquaculture industry. Sex determination in the widely farmed Nile tilapia (*Oreochromis niloticus*) is complex, involving genomic regions on at least three chromosomes (chromosomes 1, 3 and 23) and interacting in certain cases with elevated early rearing temperature as well. Thus, sex ratios may vary substantially from 50%.

**Results:**

This study focused on mapping sex-determining quantitative trait loci (QTL) in families with skewed sex ratios. These included four families that showed an excess of males (male ratio varied between 64% and 93%) when reared at standard temperature (28°C) and a fifth family in which an excess of males (96%) was observed when fry were reared at 36°C for ten days from first feeding. All the samples used in the current study were genotyped for two single-nucleotide polymorphisms (rs397507167 and rs397507165) located in the expected major sex-determining region in linkage group 1 (LG 1). The only misassigned individuals were phenotypic males with the expected female genotype, suggesting that those offspring had undergone sex-reversal with respect to the major sex-determining locus. We mapped SNPs identified from double digest Restriction-site Associated DNA (ddRAD) sequencing in these five families. Three genetic maps were constructed consisting of 641, 175 and 1,155 SNPs from the three largest families. QTL analyses provided evidence for a novel genome-wide significant QTL in LG 20. Evidence was also found for another sex-determining QTL in the fifth family, in the proximal region of LG 1.

**Conclusions:**

Overall, the results from this study suggest that these previously undetected QTLs are involved in sex determination in the Nile tilapia, causing sex reversal (masculinisation) with respect to the XX genotype at the major sex-determining locus in LG 1.

**Electronic supplementary material:**

The online version of this article (doi:10.1186/s12864-015-1383-x) contains supplementary material, which is available to authorized users.

## Background

In most fish species, the sex chromosomes are still in early stages of differentiation compared to mammals, and do not show distinct differences in length or gene content [1]. Instances of both XX/XY male heterogametic and WZ/ZZ female heterogametic sex-determining systems can be found in fish, while the fact that spontaneous sex-reversed XX males are generally fully fertile indicates that sex-determining regions can also be located on autosomes. Even though the YY genotype is lethal in mammals, YY and WW genotypes are viable in most fish species indicating that the gene content of the Y and W chromosomes are very similar to that of their X and Z counterparts respectively [2,3]. Generally an even sex ratio can be expected in species with genetic sex determination. However, departures from an equal sex ratio have been observed both in population and family studies, stressing the complexities of sex-determination in fish. Distorted population or family sex ratios are likely to be due to hormonal effects, complex genetic sex determination or interaction between genetic and environmental factors [4,5]. Environmental effects on sex ratios may vary even within species [6].

Many species of farmed fish exhibit sexual dimorphism in a range of traits of commercial importance, including growth rate or age at maturity, stimulating research to clarify the sex determining system of such fish with the objective of the production of mono-sex stocks for the aquaculture industry. *Oreochromis niloticus* (Nile tilapia) is one of the most important farmed species with a production exceeding 3.4 million tonnes in 2012 [7]. Intensive commercial production generally requires all-male stocks, not only because males grow faster but also to avoid uncontrolled reproduction before harvest.

Current evidence suggests that *O. niloticus* possesses a complex sex determination system comprising an XX/XY male heterogametic system with other genetic and environmental factors (principally temperature). The major sex-determining region has been previously located on linkage group (LG) 1 [8] and fine mapped in a region of approximately 1.2 Mb [9].

Frequent departures from equal sex ratio have also been observed where the temperature was not high enough to affect sexual differentiation, and it has been postulated that these departures are caused by other loci, potentially including those in LG 3 (the location of the WZ/ZZ sex-determining locus; [10]) and LG 23 [11-16]. Interestingly crosses between putative YY males and XX females often give less than 100% male progeny predicted from a simple XX/XY system: some such crosses give close to 100% males, while others give lower proportions of males, but still significantly higher than the 50% expected from XY males [17]. Many of the studies on sex determination in *O. niloticus* have been carried out on fish derived from Lake Manzala in Egypt, the subject of the present study, and it is clear that both non-LG 1 genes and temperature affect sex ratios in at least some families in this population.

Temperature can affect sex ratios in *O. niloticus*, with rearing temperatures above 34°C during sexual differentiation having masculinising effects [18]. Male ratios in elevated temperature-treated *O. niloticus* are strongly dependent both on the population and on the parental animals [19,20]. Family-specific quantitative trait loci (QTL) involved in sex reversal due to temperature have been identified in LG 1, 3 and 23 in genetically all-female families, coinciding with known sex-determining regions of *O. niloticus* [21]. Those studies [20,21] were carried out on a sub-population of the Stirling Lake Manzalla-derived population.

In a previous study [9] we applied a genotyping by sequencing approach using SNPs screened by Restriction-site Associated DNA (RAD) sequencing [22] in order to scan for sex-determining QTL in *O. niloticus* families of balanced sex ratios. No sex-determining region apart from the known one in LG 1 was detected. Notably, the only misassigned individuals in both the mapping families and other samples used for validation (7 samples out of the 351) were phenotypic males with the LG 1 genotype expected of females, suggesting that they had undergone sex reversal.

In the current study we extended the search for sex-determining QTLs by analysing segregating SNP polymorphisms in five families of *O. niloticus* exhibiting pronounced skewed sex ratios. Rather than using standard RAD to screen for a highly redundant set of informative SNPs within pedigrees, we employed a variant of this technology; double digest RAD (ddRAD; [23]), which allowed a subset of the RAD loci to be surveyed more straightforwardly and more economically. We identified one QTL in LG 20 that conferred masculinisation to fish classified as XX females by their LG 1 genotype, while also finding evidence for an additional second sex- determining QTL in LG 1.

## Results

### Sex ratios and LG 1 SNP analyses

The sex ratios of families 1–4 (Table 1) deviated from the expected 1:1 (*P* < 0.001). The sex ratio of the control group in family 5 did not deviate from the expected 1:1 (*P* > 0.3) while the treated group showed significant deviation, giving 96% males (*P* < 0.001). The only misassigned offspring in all five families concerning the genotype for SNPs rs397507167 and rs397507165 were males appearing with the female expected genotype (Table 2).

**Table 1:** **Fish samples used for ddRADseq libraries**

**ID**	**Sire strain**	**Dam strain**	**Sex ratio (total males/females)**	**Analysed males**	**Analysed females**	**Total analysed fish**
Family 1	Red^†^	Clonal	64% (87/49)	54	44	98
Family 2	Red^†^	Clonal	72% (120/46)	36	29	65
Family 3	Red^†^	Clonal	93% (206/15)	28	10	38
Family 4	Red^†^	Clonal	92% (404/36)	43	8	51
Family 5 (28°C)	Red^†^	Wild^*^	55% (25/20)	28	22	50
Family 5 (36°C)	Red^†^	Wild^*^	96% (66/4)	66	4	70

^*^“Wild” refers to wild-type coloration; ^†^“red” refers to red body colour, which is controlled by a single gene.

**Table 2:** **Genotypic information of samples used for ddRAD Libraries for LG 1 SNPs** ***Oni*** **23063 and** ***Oni*** **28137 (NCBI dbSNP accession rs397507167 and rs397507165 respectively)**

	**Female expected genotype**		**Male expected genotype**	
**ID**	**Males**	**Females**	**Males**	**Females**
Family 1	21	44	33	0
Family 2	9	29	27	0
Family 3	14	10	14	0
Family 4	11	8	32	0
Family 5 (28°C)	8	22	20	0
Family 5 (36°C)	28	4	38	0

### ddRAD sequencing

**Figure 1:**
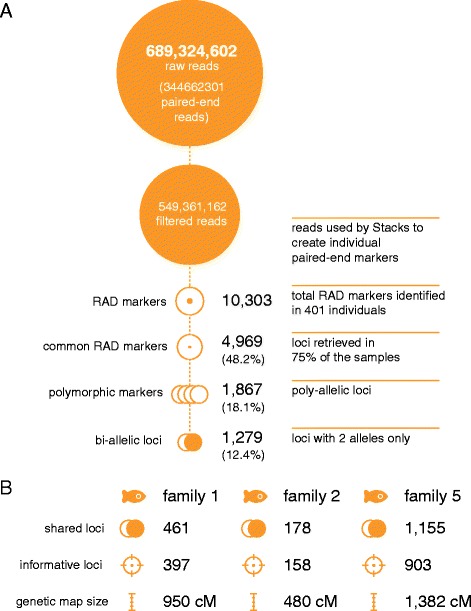
**Sequencing and polymorphic marker summary. (A)** Details of the number of reads before and after filters (orange disk) followed by the reconstructed number of RAD markers and polymorphic RAD markers (orange circles). **(B)** Genetic map reconstruction. Number of shared polymorphic markers within each family, the number of informative loci and final genetic map size.

In total, 689,324,604 raw reads (100 bases long) were produced (344,662,302 paired-end reads, EBI SRA study ERP004077). After removing low quality sequences, ambiguous barcodes and orphaned paired-end reads, 79.7% of the raw reads were retained (549,361,162 reads). In total 10,303 unique RAD-tags were retrieved (Figure 1A). The number of reads and RAD-tags for each sample are reported in Additional file 1: Table S1.

### Genetic maps

In order to maximise the number of informative markers and minimise the amount of missing or erroneous data, we used SNP markers retrieved in at least 75% of the samples in each family, and carrying one or two SNPs. Genetic maps 1, 2 and 3 were constructed from offspring of families 1, 2 and 5 respectively (Figure 1B). The linkage groups were named according to the Broad Institute of MIT and Harvard genome assembly Orenil1.1 (NCBI Assembly GCA_000188235.2). The genetic map derived from family 5 proved to be the most comprehensive, since it was derived from an outbred cross, and consisted of 1,155 SNPs (642 female-specific; 640 male-specific; 903 informative ones), grouped in 23 linkage groups that corresponded to 22 different chromosomes (the number expected from the karyotype). The total length was 1,382 cM (Table 3, Additional file 2: Table S2).

**Table 3:** **Genetic map based on offspring from high temperature-treated family**

**Linkage group**	**No. of markers**	**No. of informative markers**	**Length (cM)**
LG 1	60	45	88.3
LG 2	57	42	30.3
LG 3	55	49	68.9
LG 4	48	38	86.8
LG 5	39	33	59.5
LG 6	61	49	102.8
LG 7	135	106	134.6
LG 8	71	58	49.5
LG 9	42	32	55.5
LG 10	26	21	46.4
LG 11	47	38	55.0
LG 12	26	20	46.4
LG 13	15	13	22.8
LG 14	57	44	39.6
LG 15	15	10	5.8
LG 16	30	21	42.5
LG 17a	22	18	27.5
LG 17b	35	23	17.2
LG 18	40	32	86.2
LG 19	75	54	41.0
LG 20	75	59	89.8
LG 22	59	48	84.8
LG 23	57	50	107.9
**Total**	**1155**	**903**	**1382**

The family 1 genetic map consisted of 641 markers (resolving 397 distinct sites), grouped in 22 linkage groups that corresponded to 19 different chromosomes. The total length was 950 cM (Additional file 3: Table S3, Additional file 2: Table S2). This map did not contain information about chromosomes 5, 12 and 15 due to a lack of informative loci on those chromosomes. The family 2 genetic map consisted of 178 markers (158 informative loci), grouped into 22 linkage groups that corresponded to 18 different chromosomes, with chromosomes 15 and 22 being represented by two markers in total that were grouped in the same linkage group. The total length of the map was 480 cM (Additional file 4: Table S4, Additional file 2: Table S2). This map did not contain information about chromosomes 5 and 23. The number of informative markers in families 1 and 2 was lower as the dams were isogenic. Missing chromosomes were not the same in maps 1 and 2.

### QTL mapping and association analysis (Families 1–4)

**Figure 2:**
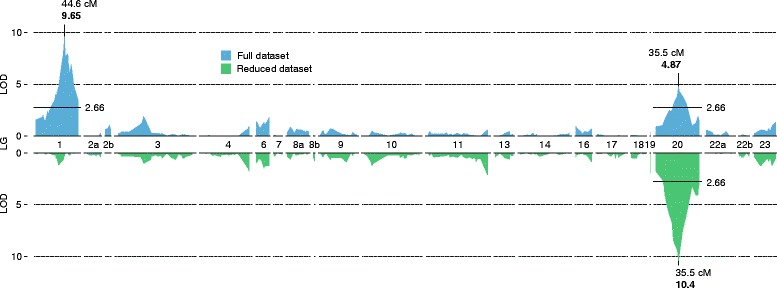
**QTL mapping in family 1.** Plots of the LOD score (sex-determining QTL search) along the linkage groups. Top graph, full dataset; bottom graph, reduced dataset (44 females and 21 males suspected to have undergone sex reversal). The numbers refer the linkage group numbers Table 3.

QTL mapping for family 1 was conducted using R/qtl. The results from the single-QTL model for binary traits provided evidence for the existence of a QTL in LG 1, in the expected position of the major sex determining region (LOD = 9.65), and a second strong QTL in LG 20 (LOD = 4.87; Figure 2). The genome-wide significance threshold for the single-QTL model had a value of LOD = 2.77 (10,000 permutations; α = 0.05). Explained variances of the above QTLs were estimated after running a multi-dimensional QTL model. The QTL on LG 1 explained approximately 40.5% of the phenotypic variance (LOD = 15.13; *P* < 10^−15^), while the QTL on LG 20 explained approximately 25% of the phenotypic variance (LOD = 10.35; *P* < 10^−11^; Table 4).

**Table 4:** **QTL mapping results in linkage group 20**

**Family**	**LOD**	**Explained phenotypic variance (%)**	**P-value**
1	10.35	24.47	< 10^−12^
2	5.8	15.6	< 10^−6^
3	-	-	-
4	-	-	-
5	2.42	6.34	0.0001

Results obtained from multidimensional QTL model (*stepwise* function; R/qtl). Families 3–4 were not informative concerning the QTL in linkage group 20.

The estimated 95% Bayesian Density Interval for the QTL on LG 20 spanned a region of 13.5 cM (34.5 – 48 cM in LG 20). In terms of physical distance the above interval corresponds to approximately 15.6 Mb (13.5- 29.1 Mb; Broad Institute of MIT and Harvard genome assembly Orenil1.1). The QTL mapping on the reduced dataset (44 females and 21 males suspected to have undergone sex reversal) detected only the QTL on LG 20 (LOD = 10.40), which explained approximately 51% (*P* < 10^−11^) of the phenotypic variance (Figure 2). The estimated 95% Bayesian Density Interval spanned a region of 3.5 cM (33.5 – 37 cM in LG 20). In terms of physical distance the above interval corresponds to approximately 11.7 Mb (12.6- 24.3 Mb; Broad Institute of MIT and Harvard genome assembly Orenil1.1). SNP marker *Oni*3161 showed the highest association for the putative sex-reversed offspring (Additional file 5: Data S1). The marker is located in the Emilin-3-like gene of *O. niloticus* (NCBI GeneID: 100703501).

QTL mapping using the single-QTL model for family 2 also identified the QTL in LG 20 in the same position as in family 1 (LOD = 2.80). The genome-wide significance threshold for the single-QTL model had a value of LOD = 2.55 (10,000 permutations; α = 0.05). The multi-dimensional QTL model estimated that the above QTL explained approximately 15.6% of the phenotypic variance (LOD = 5.8; *P* < 10^−6^; Table 4).

The Fisher’s exact tests confirmed significant deviations (*P* < 10^−14^) in terms of allelic association in SNP marker *Oni*3161 for offspring with the female expected genotype in the major sex-determining region in LG 1, while the corresponding testing for offspring with the male expected genotype was non-significant (*P* > 0.9; Table 5).

**Table 5:** **A. Allelic combinations between SNP markers of highest association with phenotypic sex on LG 1 (rs397507167) and LG 20 (** ***Oni*** **3161) for offspring in families 1 and 2; B. Allelic combinations between SNP markers of highest association with phenotypic sex on LG 1 (rs397507167) and LG 20 (** ***Oni*** **3161) for offspring in the temperature treated family (treated group 36°C)**

**A.**	**LG1: G/G**		**Fisher’s exact test**
	**Female expected genotype**		
LG20: C/C	Female: 60	Male: 0	P < 10^−14^
LG20 :C/T	Female: 12	Male: 27	
	**LG1: G/A**		
	**Male expected genotype**		
LG20: C/C	Female: 0	Male: 33	P > 0.9
LG20 :C/T	Female: 0	Male: 22	
**B.**	**LG1: G/G**		
	**Female expected genotype**		
LG20: C/C	Female: 4	Male: 8	P = 0.014
LG20 :C/T	Female: 0	Male: 20	
	**LG1: G/A**		
	**Male expected genotype**		
LG20: C/C	Female: 0	Male: 20	P > 0.9
LG20 :C/T	Female: 0	Male: 18	

The association analysis on offspring from families 1 and 2, adjusted for family effect, in addition to markers from LG 1, identified *Oni*3161 (*P* < 10^−6^) on LG 20 as significantly associated with phenotypic sex. Although no DNA from the sires of families 3 and 4 was available for analysis, all of the progeny in families 3 and 4 were heterozygous for this SNP marker, inferring that the male was homozygous for the T allele (see Table 5) and suggesting that homozygosity at the associated QTL was responsible for the high proportion of males in these families.

### High temperature-treated family (family 5)

QTL mapping for family 5 was conducted using both R/qtl and GridQTL. Only male-informative SNPs provided evidence for existence of sex-determining QTL. In the R/qtl analysis, the results from the single-QTL model for binary traits provided evidence for the existence of a QTL in LG 1, in the expected position of the major sex-determining region (LOD = 9.66). The genome-wide significance threshold for the single-QTL model had a value of LOD = 3.06 (10,000 permutations; α = 0.05).

The multidimensional QTL model provided evidence for the existence of a second, weaker QTL on LG 20. In this model, the QTL on LG 1 explained approximately 28% of the phenotypic variance (LOD = 9.37; *P* <10^−9^), while the LG 20 QTL explained approximately 6% of the phenotypic variance (LOD = 2.42; *P* < 0.001; Table 4).

In the GridQTL analysis using the half-sib regression model for single-QTL and treatment as a fixed effect, only the QTL on LG 1 (F = 53.62) was significant at the genome-wide threshold, while the QTL on LG 20 (F = 10.68) was significant only at the chromosome-wide level (α = 0.01). The genome-wide significance threshold, estimated using 10,000 permutations, had a value of F = 12.28 (α = 0.05). The two-QTL model indicated the possible existence of an additional QTL on LG 1 (F = 17.34; comparison of the 2-QTL model with the single QTL model). The above QTL was located on the 11th cM, with the peak approximately 3.4 Mb (according to Broad Institute of MIT and Harvard genome assembly Orenil1.1) distant from the aromatase gene *cyp19a1*. The Fisher’s exact tests confirmed significant deviations in terms of allelic combinations both in the case of the SNP marker with the highest association in LG 20 (*Oni*3161; *P* = 0.014) and in the case of the SNP marker *Oni*10909 (located on the QTL detected at the proximal end of LG 1; *P* = 0.004) for offspring with the female-expected genotype in the major sex determining region (Additional file 5: Data S1). The corresponding testing for offspring with the male expected genotype was non-significant (*P* > 0.95).

## Discussion

Previous studies emphasised the complexities of the *O. niloticus* sex-determining system by identifying sex-determining QTL in different chromosomes [8,12,15,24]. At the same time temperature also affects sex ratio, possibly interacting with genetic factors [25] and family-specific QTL involved in temperature induced sex reversal of genotypic females have been detected [21]. Additionally, the results of Ezaz *et al.* [26] suggested that some genetic factors might cause sex reversal in both directions, *i.e.* some families showed departures from both all-male and all-female sex ratios, while others showed no departures in either direction. All the above explain to a certain degree the observed departures from equal sex ratio that a simple XX/XY sex-determining system would suggest.

The main limitation of the previous studies that tried to detect sex-determining regions in *O. niloticus* was the limited number of genetic markers available, mainly concentrating on linkage groups 1, 3, and 23. However, since departures from equal sex ratio are not observed in all crosses, the usage of suitable crosses is also a necessary prerequisite for mapping sex-determining QTL other than LG 1 in *O. niloticus*, and in particular previously undetected QTL. In our previous study [9] we developed a high-resolution map of *O. niloticus* using families with balanced sex ratios, from which only the major sex-determining region on LG 1 was detected. In this study, we applied ddRAD in families with skewed sex ratios, in which we expected that additional QTL would be affecting sex determination.

All the samples used in the current study were genotyped for two SNP markers located in the expected major sex-determining region in LG 1 (rs397507167, rs397507165) and one microsatellite (*UNH995*) that is also tightly linked to this region [8]. The only misassigned individuals were phenotypic males appearing with the expected female genotype, as seen in our previous study [9]. This indicates sex reversal of genotypic (LG 1 XX) females.

The detected QTL in LG 20 in the current study provides the first evidence for a sex-determining region in this chromosome with possible involvement in sex reversal. The possibility that the above QTL is involved in sex reversal is also strengthened by the fact that its LOD score more than doubled in the analysis of family 1 (LOD whole dataset: 4.84; LOD reduced dataset: 10.40) when only the females and the putative sex-reversed males were used. A QTL on LG 20 was also identified in the analysis of family 2 offspring. Although the significance was just above the genome-wide threshold when the single-QTL model was used (QTL LOD = 2.80; significance threshold LOD = 2.55), using the multi-dimensional QTL model provided clear evidence of its existence (LOD = 5.80). The difference in the estimated QTL values between family 1 and 2 is most probably due to the lower statistical power of the analysis of family 2 due to its smaller informative sample size, requiring the usage of more elaborate models. Models that take into account the existence of a major QTL (major sex-determining region) or ones that test for existence of multiple QTLs simultaneously reduce the residual variation - providing higher power in the analysis for detecting additional QTLs at least of moderate effect [27].

The inferred paternal genotypes (family 3–4) were not informative concerning the most probable location for the QTL on LG 20 (*Oni*3161). No QTL apart from the expecting one on LG 1 (major sex-determining region) was identified in those families. It seems likely that the sires of families 3 and 4 were homozygous for the masculinizing allele of the LG 20 QTL and thus non-informative for this analysis. However, the association analysis conducted on offspring from four families (1–4) provided evidence for the existence of a genome-wide significant sex-determining region on LG 20.

The QTL on LG 20 was found to be significant only at the chromosome-wide level in the high temperature-treated family (family 5). It has to be stressed though, that the data from the high temperature-treated cross was not as informative as that from family 1 or family 2. The fact that the high temperature-treated group contained almost all male progeny, with only 4 females, forced us to analyse both treated and control groups simultaneously, adding the factor treatment as a fixed effect (treated; untreated). Crosses involving high temperature treatment of genetic all-female progeny [21] would have been more informative. However, the fact that a signal in terms of association with sex was detected at the same genomic location in families 1, 2 and 5 would suggest that the same underlying mechanism is involved in both cases, causing sex reversal of genotypic females.

Interestingly the two-dimensional QTL scan in the temperature-treated family (family 5) provided evidence for another QTL at the beginning of LG 1, which could also be implicated in sex reversal. The location of the above QTL is in proximity (approximately 3.4 Mb) to the *cyp19a1* gene. *Cyp19a1* is the enzyme that catalyses the irreversible conversion of androgens into oestrogens and has been shown to be suppressed at masculinising temperatures [4]. Methylation in the promoter of *cyp19a1* was shown to be involved in temperature-dependent sex-determination in *Dicentrarchus labrax* (European sea bass; [28]). However, it has to be stressed that in the current study, we cannot exclude the possibility of this QTL on LG 1 being a false positive.

*O. niloticus* presents a unique combination of properties for the study of the genetic basis of elevated temperature effects on sex determination. Although temperature effects on sex determination have been observed in many species of fish and reptiles, intraspecific polymorphism for this trait has been found in only a few species. In addition to tilapia, this has been documented in *Menidia menidia* (Atlantic silverside; [29]), *Alligator mississipiensis* (American alligator), *Chelydra serpentine* (common snapping turtle), *Chrysemys picta* (painted turtle; [30]) and *Lepomis macrochirus* (bluegill sunfish; [31]). Such polymorphism allows approaches such as mapping the responsible loci, which in the case of *O. niloticus* is facilitated by the ease with which it can be bred and reared in captivity and the supporting genomics resources. To our knowledge, mapping studies on genes controlling temperature effects on sex determination have only been carried out in the Nile tilapia: the results emerging from these studies map loci controlling variation in temperature effects (LG 1, LG 3, LG 23 –[21]; LG 20 – present study) to genomic regions that have been demonstrated to have strong effects on sex determination at standard rearing temperatures (*i.e.*, the same loci involved in “genetic” sex determination).

## Conclusions

In summary, the current study provides new insights about novel sex-determining regions in LG 20 and LG 1 involved in *O. niloticus* sex reversal relative to the main sex-determining region in LG 1. In the case of the QTL on LG 20, strong evidence of its existence is supported by the results from three different families. More informative crosses, especially concerning the study of temperature effects, would shed more light concerning the magnitude of the detected QTLs. It seems likely that these (and maybe other) QTL could account for the unexpected sex ratios encountered during the development of YY *O. niloticus*, and the difficulties in producing high percentages of males in progeny of YYs with only phenotypic sex ratio as a tool. The outcome of the current study stresses the complexities of the *O. niloticus* sex determining system and the potential offered by High-Throughput Sequencing platforms like ddRAD-seq in unveiling those complexities.

## Methods

### Ethic statement

All working procedures complied with the UK Animals Scientific Procedures Act [32].

### Sample collection and preparation

The fish used in this study were reared in the Tropical Aquarium Facilities of the Institute of Aquaculture at the University of Stirling. They originated from a population that was established in 1979 from fish taken from Lake Manzala, Egypt (31°16′N, 32°12′E). Fish were reared in recirculating water systems at 27-28°C, and fed on commercial trout diet (Trouw Aquaculture Nutrition, UK; manufacturer Skretting, UK). To set up the families used in this study, mature females were held in glass aquaria and eggs were manually stripped following ovulation. Milt was manually stripped from male fish and used to fertilise the eggs in vitro. Eggs were incubated in down-welling incubators until the larvae had absorbed the yolk sac. Fry from families 1–4 were then transferred to tanks in recirculating systems and reared for 3–4 months before being killed and sexed by microscopic examination of the gonads [33]. A sample of fin tissue was taken and fixed in 100% ethanol for DNA extraction. Family 5 was split at yolk sac absorption: one group of 80 fry was reared at 36°C for ten days in a static 5 L tank to induce sex reversal [20], then at 28°C in a recirculating system until sexing, while a control group (80 fry) was reared at 28°C throughout. The survival of the two groups was 88% and 91% respectively. Subsequent rearing and sexing was as for families 1–4.

Offspring from families 1–4 showed significant deviations from equal sex ratio with 64% to 93% being male (*P* < 0.01; Table 1). Offspring were selected from families 1–2 in order to have a close to equal representation of males and females for preparing the ddRAD libraries. In the case of families 3 and 4, DNA was available from only eight and ten females respectively (Table 1). A subset of the above families (46 offspring from each family) was previously genotyped using microsatellites: one of the paternal alleles of LG 1 *UNH995* (236 bp long) was always associated with male progeny, while the other was associated with male and female progeny [34]. The dams of the above families originated from an isogenic XX line [9,35], while sires were from the outbred red body coloured sub-population. One ddRAD library was prepared from family 1, a second from families 2–4, and a third from offspring of family 5 (comprising both the control and the temperature-treated groups, total 120 offspring; Table 1).

### ddRAD library preparation and sequencing

The ddRAD library preparation protocol followed a modified version of the methodology described by Peterson *et al.* [23]. Each sample (0.1 μg DNA) was digested at 37°C for 40 minutes with *Sbf*I (recognising the CCTGCA|GG motif) and *Sph*I (recognising the GCATG|C motif) high fidelity restriction enzymes (New England Biolabs, UK; NEB), using 6 U of each enzyme per microgram of genomic DNA in 1× Reaction Buffer 4 (NEB). The reactions (5 μL final volumes) were then heat inactivated at 65°C for 20 minutes. Individual-specific combinations of P1 and P2 adapters, each with a unique 5 or 7 bp barcode, were ligated to the digested DNA at 22°C for 60 minutes by adding 1 μL *Sbf*I compatible P1 adapter (25 nM), 0.7 μL *Sph*I compatible P2 adapter (100 nM), 0.06 μL 100 mmol/L rATP (Promega, UK), 0.95 μL 1× Reaction Buffer 2 (NEB), 0.05 μL T4 ligase (NEB, 2 × 10^6^ U/mL) and reaction volumes made up to 8 μL with nuclease-free water for each sample. Following heat inactivation at 65°C for 20 minutes, the ligation reactions were slowly cooled to room temperature (over 1 hour) then combined in a single pool (for one sequencing lane) and purified. Size selection (300–600 bp) was performed by agarose gel separation and followed by gel purification and PCR amplification. A total of 100 μl of the amplified libraries (13–14 cycles) was purified using an equal volume of AMPure beads. After eluting into 20 μL EB buffer (MinElute Gel Purification Kit, Qiagen, UK), the libraries were ready for sequencing. The two large pedigree libraries (Families 1 and 5) were sequenced at Edinburgh Genomics Facility, University of Edinburgh, on two lanes of an Illumina HiSeq 2500 UK (v3 chemistry, 100 base paired-end reads). The smaller pedigrees (Families 2–4) were sequenced at the University of Stirling using two runs of an Illumina MiSeq (v2 chemistry, 150 base paired-end reads). Raw reads were processed using RTA 1.18.54 (Illumina).

### Genotyping ddRAD alleles

Reads of low quality (QC values under 30), missing the expected restriction site or with ambiguous barcodes were discarded. Retained reads were sorted into loci using a reference-based assembly (Broad Institute of MIT and Harvard genome assembly Orenil1.1, based on the same isogenic line used as the dams for families 1–4 in the current study) and genotyped using Stacks software 1.13 [36]. The likelihood-based SNP-calling algorithm [37] implemented in Stacks evaluates each nucleotide position in every RAD-tag of all individuals, thereby statistically differentiating true SNPs from sequencing errors. A minimum stack depth of at least 20 and a maximum of 2 mismatches were allowed in a locus in an individual, with an additional mismatch allowed between individuals. Polymorphic ddRAD-tags may contain more than one SNP, but the vast majority (over 99%) showed only two allelic versions; ddRAD-tags with more than two alleles were excluded. All samples used for constructing the ddRAD libraries were also genotyped for SNP markers *Oni*23063 and *Oni*28137 within the major sex determining region on LG 1 (NCBI dbSNP accession rs397507167 and rs397507165 respectively) previously described in Palaiokostas *et al.* [9].

### Genetic map construction

SNP markers were initially tested for segregation distortion using the *chisq* module of TMAP [38]. Three genetic maps were constructed based on the offspring of families 1, 2 and 5 using R/Onemap [39]. Female offspring numbers for families 3 and 4 were too small for QTL mapping. Recombination rates, allocation of markers into linkage groups and ordering were conducted using R/Onemap (functions: *rf.2pts*, *group*, *ug*, *rcd*, *record*). This package uses Hidden Markov Models (HMM) algorithms for outbred species while implements, in parallel, the methodology described in Wu *et al.* [40] for calculating the most probable linkage phase. Linkage groups were formed using a minimum logarithm of the odds (LOD) value of 5. Map distances were calculated in centiMorgans (cM) using the Kosambi mapping function [41].

### QTL mapping and association analysis

#### Families 1–4 (outbred male x clonal female)

QTL analysis for families 1 and 2 was performed using R/qtl [27]. In families 1 and 2, with the dam originating from a clonal line and by inferring the most probable phase of the genetic markers of the sire, the cross had the same properties as a backcross and was analysed as such. Models following single and multidimensional approach for detecting QTL were used (R/qtl functions: *scanone*, *addqtl*, *scantwo*, *fitqtl*, *stepwiseqtl*). Permutation tests (10,000 permutations) were conducted in order to correct for the multiple testing. The analysis of the largest family (family 1) comprised two sequential steps. An initial QTL analysis was conducted using all the offspring, followed by analysis of the female offspring (44 offspring) together with the male offspring (n = 21) suspected of having undergone sex reversal, based on their female expected genotype at SNP markers in the major sex-determining region. Fisher’s exact tests were used to test for significance between allelic combinations in different loci.

Association analysis was performed on the combined dataset from families 1–4 using the R package SNassoc [42]. A Bernoulli generalised linear model was applied in order to test the magnitude of association between the SNP marker genotypes and phenotypic sex, adjusting for family effect (function *WGassociation*). The Bonferroni correction was used to correct for multiple testing.

### Family 5 (outbred cross; treated with elevated temperature)

The QTL analysis was conducted using both R/qtl and GridQTL [43]. For R/qtl the cross was considered as a ‘pseudo’ backcross, analysing male and female informative markers separately. The half-sib regression model was used in the analysis conducted using GridQTL using treatment (temperature treated or control group) as a fixed effect. Models following single and multidimensional approach for detecting QTL were used (GridQTL: scanning for single and two-QTL simultaneously). Permutation tests (10,000 permutations) were conducted in order to correct for the multiple testing, while in the case of GridQTL two levels of significance are reported based on chromosome- or genome-wide thresholds, with the detected QTL being referred to as suggestive or significant respectively [44-46]. Fisher’s exact tests were used to test for significance between allelic combinations in different loci.

### Data access

The raw sequence data from this study have been submitted to the EBI Sequence Read Archive (SRA) study ERP004077. The SNP marker *Oni*3161 and *Oni*10909 were deposited on at the NCBI dbSNP with the assay ID ss1026566023, ss1026566024 respectively.

## Additional files

Additional file 1: Table S1. Samples origin and barcode. Details each sample used: sample ID, family, gender, barcode used, number of raw reads paired-ended and number of RAD-tags. Additional file 2: Table S2. Genetic maps. Ordered markers: marker ID, linkage group and position (cM). Additional file 3: Table S3. Genetic map based on offspring from family 1. Additional file 4: Table S4. Genetic map based on offspring of family 2. Additional file 5: Data S1. Marker sequences. Details of SNP alleles and RAD-tag allele sequences of the markers.

## Abbreviations

ddRAD: Double digest restriction-site associated DNA
SNP: Single nucleotide polymorphism
QTL: Quantitative trait locus
LG: Linkage group
